# miRNome Profiling and Functional Analysis Reveal Involvement of hsa-miR-1246 in Colon Adenoma-Carcinoma Transition by Targeting *AXIN2* and *CFTR*

**DOI:** 10.3390/ijms23042107

**Published:** 2022-02-14

**Authors:** Rokas Lukosevicius, Simonas Juzenas, Violeta Salteniene, Ugne Kulokiene, Justina Arstikyte, Georg Hemmrich-Stanisak, Andre Franke, Alexander Link, Paulius Ruzgys, Saulius Satkauskas, Henrikas Pauzas, Tadas Latkauskas, Gediminas Kiudelis, Francesc Balaguer, Juozas Kupcinskas, Jurgita Skieceviciene

**Affiliations:** 1 Institute for Digestive Research, Academy of Medicine, Lithuanian University of Health Sciences, 44307 Kaunas, Lithuania; rokas.lukosevicius@lsmuni.lt (R.L.); simonas.juzenas@lsmuni.lt (S.J.); violeta.salteniene@lsmuni.lt (V.S.); ugne.kulokiene@lsmuni.lt (U.K.); Justina.aristikyte@lsmuni.lt (J.A.); 2 Institute of Clinical Molecular Biology, Christian-Albrechts-University of Kiel, 24105 Kiel, Germany; g.hemmrich-stanisak@ikmb.uni-kiel.de (G.H.-S.); a.franke@mucosa.de (A.F.); 3 Department of Gastroenterology, Hepatology and Infectious Diseases, Otto-von-Guericke University, 39106 Magdeburg, Germany; Alexander.Link@med.ovgu.de; 4 Biophysical Research Group, Faculty of Natural Sciences, Vytautas Magnus University, 44248 Kaunas, Lithuania; paulius.ruzgys@vdu.lt (P.R.); saulius.satkauskas@vdu.lt (S.S.); 5 Department of Surgery, Lithuanian University of Health Sciences, 44307 Kaunas, Lithuania; henrikas.pauzas@lsmuni.lt (H.P.); tadas.latkauskas@lsmuni.lt (T.L.); 6 Department of Gastroenterology, Lithuanian University of Health Sciences, 44307 Kaunas, Lithuania; gediminas.kiudelis@lsmuni.lt; 7 Department of Gastroenterology, Hospital Clínic Barcelona, Centro de Investigación Biomédica en Red en Enfermedades Hepáticas y Digestivas (CIBEREHD), IDIBAPS (Institut d'Investigacions Biomèdiques August Pi i Sunyer), University of Barcelona, 08036 Barcelona, Spain; fprunes@clinic.cat

**Keywords:** colorectal cancer, adenomatous polyps, microRNA, isomiR, small RNA-seq

## Abstract

Regulatory changes occurring early in colorectal cancer development remain poorly investigated. Since the majority of cases develop from polyps in the adenoma-carcinoma transition, a search of early molecular features, such as aberrations in miRNA expression occurring prior to cancer development, would enable identification of potentially causal, rather than consequential, candidates in the progression of polyp to cancer. In the current study, by employing small RNA-seq profiling of colon biopsy samples, we described differentially expressed miRNAs and their isoforms in the adenoma-carcinoma transition. Analysis of healthy-adenoma-carcinoma sequence in an independent validation group enabled us to identify early deregulated miRNAs including hsa-miR-1246 and hsa-miR-215-5p, the expressions of which are, respectively, gradually increasing and decreasing. Loss-of-function experiments revealed that inhibition of hsa-miR-1246 lead to reduced cell viability, colony formation, and migration rate, thereby indicating an oncogenic effect of this miRNA in vitro. Subsequent western blot and luciferase reporter assay provided evidence of hsa-miR-1246 being involved in the regulation of target *AXIN2* and *CFTR* genes’ expression. To conclude, the present study revealed possible involvement of hsa-miR-1246 in early colorectal cancer development and regulation of tumor suppressors AXIN2 and CFTR.

## 1. Introduction

Colorectal cancer (CRC) is currently one of the most prevalent cancer types worldwide and is the second leading cause of cancer-related mortality [1]. Generally, from 70% to 90% of CRC cases arise from the traditional adenoma-carcinoma transition, where conventional adenomas (polyps) progress by the sequential accumulation of genetic mutations and chromosomal instability causing microsatellite stable adenocarcinomas [2]. In the adenoma-carcinoma progression, the above-mentioned alterations inactivate tumor-suppressor genes and activate oncogenes leading to cancer stem cell formation, which is essential for CRC initiation, growth, and maintenance [2,3]. Multiple genetic, transcriptional and functional studies have identified numerous coding genes that affect viability, growth and migration of CRC cells [4]. These findings, besides providing important aspects of CRC pathogenesis, also contributed to the development of novel therapeutic targets (e.g., anti-VEGF and anti-EGFR monoclonal antibodies [5]) and patient stratification strategies [6]. However, the effectiveness of these approaches has been proven only in the subsets of patients, and thus disease management success remains poor and requires new defined molecular features. Therefore, investigating the regulatory mechanisms that occur prior to cancer stem cell development, at a polyp stage, is a promising field of CRC research for possible therapeutic agents, preventive treatment, and diagnostic biomarkers.

In addition to the genetic variability and differential expression of coding genes among tumor cells, noncoding RNAs and specific epigenetic marks further contribute to functional tumor heterogeneity [3]. For example, microRNAs (miRNAs) are known to regulate different aspects of tumorigenesis, from tumor initiation to the maintenance of established tumors [7]. These small RNAs mediate translational repression of messenger RNAs (mRNAs) through 3’UTR-specific antisense interactions [8] and, based on their function in cancerogenesis, can act either as oncogenes or tumor suppressors [9]. Although miRNAs are annotated and cataloged as single defined sequences, alternative processing choices (incl. alternative Drosha and/or Dicer mediated cleavage, non-templated nucleotide addition and RNA editing) along the miRNA biogenesis pathway can generate multiple miRNA isoforms (isomiRs) which can expand the regulatory repertoire of miRNA genes and either enhance or reduce their cancerogenicity [8,10]. It has been shown that miRNAs (incl. their isoforms) are deregulated in nearly all cancer types, including CRC [11,12,13,14,15,16], and might be used as biomarkers [17] as well as therapeutic targets [18] in various diseases. Therefore, molecular profiling and functional examination of aberrantly expressed miRNAs along the adenoma-carcinoma transition is needed to further understand the pathogenesis of CRC and to identify putative targets for treatment or even diagnostics of the disease.

In this study, using small RNA-seq profiling of colon biopsy samples from healthy controls (HC) and patients with CRC or colon adenomatous polyps (AP), we described differentially expressed miRNAs and their isoforms in the adenoma-carcinoma transition. In addition to this, by utilizing RT-qPCR validation in the independent group of patients, we reported a robust, early, and gradual deregulation of hsa-miR-1246 and hsa-miR-215-5p along HC, AP, and CRC groups as well as up-regulation of hsa-miR-1246 in plasma samples of patients with CRC. Finally, by using functional assays, we showed that hsa-miR-1246 exhibits an oncogenic effect in vitro and provided evidence of its involvement in the direct regulation of *AXIN2* and *CFTR* gene expression.

## 2. Results

### 2.1. Small RNA-Seq Defines Altered Expression of miRNAs and isomiRs along Adenoma-Carcinoma Sequence

**Figure 1:**
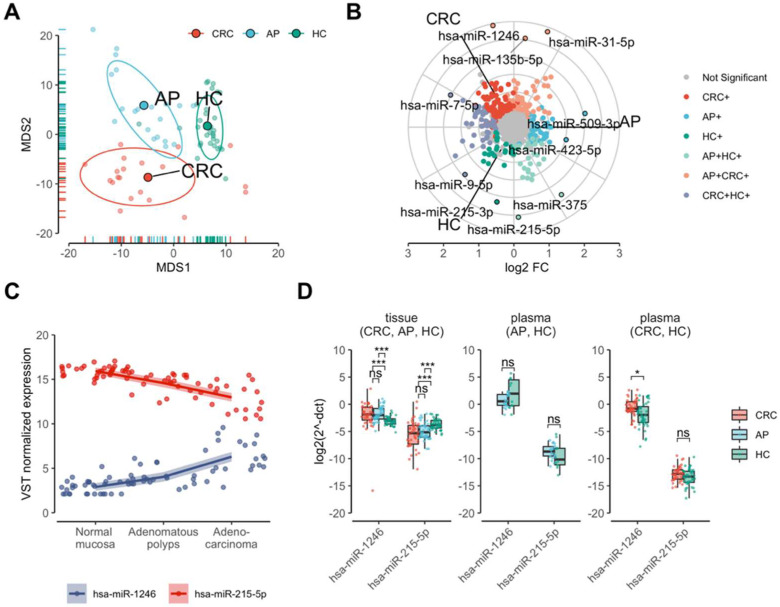
Expression profiling of miRNAs in precancerous and cancerous tissues of the colon. (**A**) MDS plot showing three clearly resolved clusters corresponding to colorectal cancer (CRC) and adenomatous polyps (AP) patients and healthy controls (HC). The analysis was performed on normalized miRNA count data using Euclidean distance. (**B**) A radar plot showing commonly and uniquely deregulated miRNAs (P_FDR_ < 0.01 and |log_2_FC| > 0.5) among CRC, AP, and HC groups (indicated by colors) in small RNA-seq data. (**C**) miRNAs highly correlating (absolute value of r_Spearman’s_ > 0.7) with the stages of healthy to adenoma-carcinoma sequence. Spearman’s rank correlation analysis was performed on variance stabilizing transformed miRNA counts. (**D**) The boxplots display expression levels (delta Ct) of hsa-miR-1246 and hsa-miR-215-5p measured by RT-qPCR in tissue and plasma samples of an independent validation cohort of HC, AP, and CRC individuals. Dots located along the box plots indicate miRNA expression level of each individual in the validation group. Due to the use of different RT-qPCR assays, the results of AP and CRC plasma samples are displayed separately. The ΔCt values were inversed in order to show true direction of the expression. Significance levels: * P_FDR_ < 0.05; *** P_FDR_ < 0.001; ns—not significant.

To evaluate whether the changes in miRNA expression occur in the transition from normal colon tissue to adenoma and from adenoma to adenocarcinoma, small RNA-Seq was performed on tissue biopsies of CRC, AP, and HC individuals. The global similarity of miRNA transcriptomes was estimated using multidimensional scaling analysis (MDS) on VST-normalized miRNA count data (see Section 4). The analysis showed three clearly resolved clusters concordant with CRC, AP, and HC groups. The AP cluster was an intermediate between CRC and HC groups. To be precise, the AP cluster showed high overlap with CRC on the first MDS (MDS1) dimension and high overlap with HC on the second MDS (MDS2) dimension (Figure 1A), which suggests transitional changes in global miRNA expression levels from normal mucosa to adenocarcinoma.

To explore whether the variability within miRNA sequences is also reflected in the adenoma-carcinoma progression, the analysis of isomiRs (variations at the 5’ and 3’ ends, nucleotide (nt) substitutions, and non-templated 3’ additions) was performed. The overall abundances of isomiR modification types were unevenly distributed (P_FDR_ < 0.05) across the groups, except for 3’ addition (Supplementary Figure S1A). Interestingly, the abundances of 3’ addition and seed shifting (5’-trimming) modifications were significantly gradually decreasing from HC to CRC groups at positions 1 and −1 (with respect to reference miRNA), respectively (Supplementary Figure S1B,C). These results show that some types of miRNA modifications were distributed in a transitional fashion, where AP tissue samples had intermediate values between CRC and HC groups.

To further define differences in miRNA and their isoform expression among CRC, AP, and HC tissues, differential expression analysis was performed. The analysis identified 208 differentially expressed miRNAs, 157 of which were up-regulated and 51 were down-regulated in CRC when compared with HC, whereas 146 miRNAs were found to be deregulated in AP compared with HC. The majority of those were up-regulated (*n* = 89) in AP and only a number of miRNAs (*n* = 57) were down-regulated. Differential expression analysis between CRC and AP identified 191 altered miRNAs, 122 of which were up-regulated and 69 were down-regulated (differential expression analysis results 1 at GEO under the accession number of GSE160432). The results of deregulated isomiRs in the analogous comparisons are provided in Differential expression analysis results 2 in the GEO Supplementary File Section under the accession number of GSE160432. The three-way differential expression analysis of both colon conditions (CRC and AP) against HC revealed that the expression levels of 60 miRNAs (such as hsa-miR-1246, hsa-miR-31-5p, and hsa-miR-135b-5p) were commonly elevated in the AP and CRC tissue samples when compared with HC. In CRC, 67 miRNAs were uniquely up-regulated, whereas in the AP alone, the expression of 37 distinct miRNAs (including hsa-miR-509-3p and hsa-miR-423-5p) were increased. Interestingly, HC and CRC had more commonly up-regulated miRNAs (*n* = 49; including hsa-miR-7-5p and hsa-miR-9-5p) compared with commonly upregulated miRNAs (*n* = 27; including hsa-miR-215-5p and hsa-miR-375) in HC and AP groups (Figure 1B; three-way differential expression analysis of miRNAs in GEO under the accession number of GSE160432).

Taken together, the results of small RNA-seq showed a high overlap between CRC and AP miRNA transcriptomes and reflected AP being the intermediate condition between CRC and HC.

### 2.2. Expression Levels of hsa-miR-1246 and hsa-miR-215-5p Are Gradually and Robustly Changing along the Healty Contol, Colon Adenoma and Adenocarcinoma Groups

To identify miRNAs that correlate with adenoma to carcinoma pathway, Spearman’s rank correlation analysis was performed by treating transitional disease stages as ordinal values. Two miRNAs (hsa-miR-1246 and hsa-miR-215-5p) highly correlated (|r_Spearman’s_| > 0.7) with the adenoma-carcinoma sequence. Gradual up-regulation of hsa-miR-1246 was determined in colorectal polyps and cancer when compared with healthy mucosa, whereas hsa-miR-215-5p was gradually down-regulated in the same sequence (Figure 1C; moderately and highly correlating miRNAs). The analysis also identified 28 moderately correlating (0.5 < |r_Spearman’s_| < 0.7) miRNAs along the HC, AP, and CRC groups, 21 of which were gradually up-regulated and seven were gradually down-regulated (Supplementary Figure S2; moderately and highly correlating miRNAs results may be found in GEO under the accession number of GSE160432).

To further evaluate whether the highly correlating hsa-miR-1246 and hsa-miR-215-5p are robustly differentially expressed along adenoma-carcinoma sequence, the RT-qPCR validation was performed in colon tissues of an independent cohort comprised of 120 individuals (CRC = 40; AP = 40; HC = 40) (Table 1). Differential expression analyses showed that both of the miRNAs were deregulated in CRC and AP tissue when compared with HC in the same direction as in the NGS data (Figure 1D).

To acquire more insights and to test whether the expression changes of these two miRNAs can be detected in the body fluids of the patients, the second validation step was performed in plasma samples of the same cohort (*n* = 120) using the RT-qPCR method. The analysis revealed that the normalized expression levels of hsa-miR-1246 were significantly (P_FDR_ < 0.05) up-regulated in CRC when compared with HC, while the expression levels of hsa-miR-215-5p were not deregulated in CRC and AP plasma samples (Figure 1D). To further determine the possible biological impact of hsa-miR-215-5p and hsa-miR-1246 in CRC pathogenesis, target gene set enrichment analysis (GSEA) was performed. In total, five Reactome pathways were significantly enriched (P_FDR_ < 0.05 and E > 1) in hsa-miR-215-5p and/or hsa-miR-1246 target genes, three out of which were responsible for “Signaling by WNT in cancer” (both miRNAs: P_FDR_ = 2.12 × 10^−3^; hsa-miR-215-5p: P_FDR_ = 0.015), “Degradation of beta-catenin by the destruction complex” (both miRNAs: P_FDR_ = 2.72 × 10^−5^), and “Binding of TCF/LEF:CTNNB1 to target gene promoters” (hsa-miR-1246: P_FDR_ = 0.024). Other two significantly enriched pathways for hsa-miR-1246 targets were the “cAMP signaling pathway” (P_FDR_ = 0.03; KEGG) and the “BDNF signaling pathway” (P_FDR_ = 0.04; WikiPathways) (Supplementary Figure S3).

### 2.3. Inhibition of hsa-miR-1246 Reduced Cell Viability, Colony Formation and Migration Rate in CRC Cells

**Figure 2:**
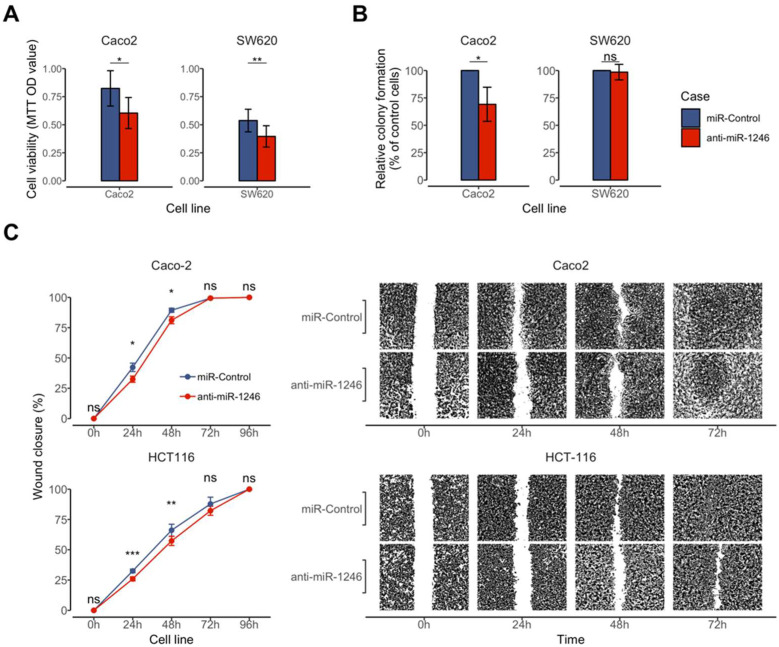
Effect of hsa-miR-1246 inhibition on cell viability, colony formation, and migration of Caco-2, HCT116, and SW620 cells. Inhibitor anti-miR-1246: (**A**) reduced cell viability in Caco-2 and SW620 cells; (**B**) reduced colony formation ability in Caco-2 cells; (**C**) lowered cell migration rates in Caco-2 and HCT116 cells 24 h and 48 h after transfection. Image magnification is ×20. Data are presented as the mean ± standard deviation (SD) of three independent experiments. Significance levels: * P_FDR_ < 0.05; ** P_FDR_ < 0.01; *** P_FDR_ < 0.001; ns—not significant. Since wounds were fully closed after 96 h, images representing this time point were not included in the figure.

Since hsa-miR-1246 showed gradual up-regulation along HC, AP, and CRC tissues and up-regulation in CRC plasma, suggesting a possible oncogenic effect, and it was selected for further functional characterization. To test this hypothesis, MTT and colony formation assays were performed to evaluate the effect of hsa-miR-1246 on cell viability and proliferation. The MTT assay revealed that inhibition of hsa-miR-1246 reduced the viability of Caco-2 (26.3%, *p* = 0.02) and SW620 (26.67%, *p* = 0.002) cells 72 h after transfection (Figure 2A). In addition, the inhibition of hsa-miR-1246 reduced colony number formed by Caco-2 cells (43.3%, *p* = 0.03) while no differences were found in SW620 cells (*p* > 0.05) (Figure 2B).

The effect of hsa-miR-1246 on CRC cell migration was examined using a wound-healing assay. Complete coverage (100%) of the gap area was reached 72 h after cell transfection with both miR-Control and hsa-miR-1246 inhibitor in the Caco-2 cell line and 96 h after transfection with miR-Control and hsa-miR-1246 inhibitor in the HCT116 cell line. Inhibition of hsa-miR-1246 significantly slowed gap covering in Caco-2 and HCT116 cells 24 h (Caco-2: 9.79%, *p* = 0.018; HCT116: 6.55%, *p* = 0.01) and 48 h (Caco-2: 8.24%, *p* = 0.04; HCT116: 8.82%, *p* = 0.007) after transfection when compared with miR-Control (Figure 2C).

Collectively, the results of the functional tests in CRC-originated cells support the potential oncogenic function of hsa-miR-1246 in CRC.

### 2.4. hsa-miR-1246 Suppresses the Expression of CFTR and AXIN2 by Directly Targeting Their 3’UTR

**Figure 3:**
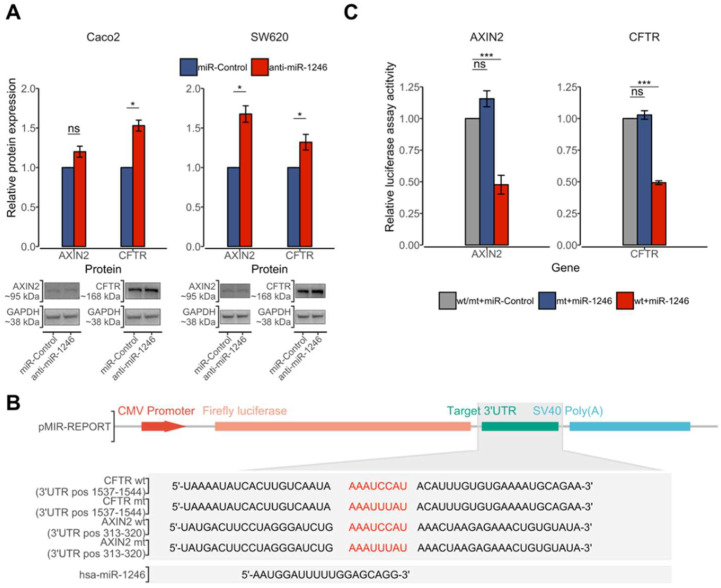
hsa-miR-1246 suppresses expression of CFTR and AXIN2 via direct 3’UTR targeting. (**A**) Effect of hsa-miR-1246 inhibition on CFTR and AXIN2 protein expression compared with negative control (miR-Control) measured at 72 h after transfection of Caco-2 and SW620 cells. Protein bands representing the signals detected by Western blot are provided at the bottom of the panel. Protein expression was normalized to the expression values of GAPDH reference protein. Data from three to four independent experiments presented as the mean of relative protein expression ± standard deviation (SD). (**B**) Representation of constructed pMIR-REPORT vectors containing wild type (wt) and mutant type (mt) 3’UTR sequences of *AXIN2* and *CFTR* genes, which are within the predicted binding sites of hsa-miR-1246. (**C**) Downregulation of *AXIN2* and *CFTR* by hsa-miR-1246 mimics. Luciferase activity of cells transfected with negative controls (miR-Control) was used as a normalization factor and was set at 100%. Data are presented as the mean ± SD of three independent experiments. Luciferase activity significantly decreased in co-transfection with hsa-miR-1246 mimic and wild-type 3’-UTR-luciferase plasmid of both *CFTR* and *AXIN2* target genes after 72 h Significance levels: * P_FDR_ < 0.05; *** P_FDR_ < 0.001; ns—not significant.

Considering that cell migration and proliferation rates were reduced by the inhibition of hsa-miR-1246, the predicted target genes were also selected based on their function and possible involvement in the WNT signaling pathway, which regulates cell fate determination, cell migration, and cell polarity [19]. More precisely, using in silico predictions, targets of hsa-miR-1246 containing 8-mer binding sites were retrieved from TargetScan database and the literature was searched for their known function in oncogenesis [20,21]. Predicted target genes for hsa-miR-1246, namely *AXIN2* and *CFTR*, met the selection criteria and were chosen for the downstream analysis of protein expression. Inhibition of hsa-miR-1246 increased levels of AXIN2 and CFTR proteins in Caco-2 and SW620 cells 72 h after transfection with anti-miR-1246. Level of CFTR protein was higher in both Caco-2 (53%, *p* = 0.02) and SW620 (32%, *p* = 0.04) cells, while AXIN2 was significantly higher only in SW620 (68%, *p* = 0.02) cells compared with the negative control (miR-Control) (Figure 3A).

To test whether the observed upregulation of AXIN2 and CFTR proteins is due to the direct interaction between hsa-miR-1246 and target (3’UTR) sequences, their 3’UTR fragments corresponding to the putative miRNA target sites (wild-type or mutated) were cloned into a luciferase vector (Figure 3B). The constructed vectors together with hsa-miR-1246 mimics or negative controls were transfected into cells. The transfection of hsa-miR-1246 mimics significantly reduced wt-CFTR and wt-AXIN2 luciferase reporter activity compared with negative miRNA control (miR-Control) (Figure 3C). The reporter activity did not change in plasmids containing mt-CFTR and mt-AXIN2 constructs after hsa-miR-1246 mimic and miR-Control transfection. These findings demonstrate that hsa-miR-1246 specifically targets *CFTR* (position 1537–1544 of 3’UTR) and *AXIN2* (position 313–320 of 3’UTR) transcripts by binding to their target sites. These findings together with the results of the functional tests in CRC cells support the hypothesis that increased levels of hsa-miR-1246 might enhance translational repression of *AXIN2* and *CFTR* genes, both of which are members of the WNT signaling pathway.

## 3. Discussion

The majority of CRC pathologies derive from a polyp in a so-called traditional adenoma-carcinoma transition, where an aberrant colon crypt evolves into an adenomatous polyp and eventually progresses to CRC [2]. This process not only involves sequential acquisition of genetic alterations, but also epigenetic aberrations such as uncontrolled expression of regulatory miRNAs [22]. For this reason, a search of early regulatory changes occurring at the polyp stage provides a possibility to identify putative candidates that are potentially causal, rather than consequential, in the progression of adenoma to cancer.

By following this logic, small RNA-seq profiling was performed on colon tissue samples from healthy individuals and patients with colon adenomas or with CRC. Subsequent exploratory analysis on the similarity structure of obtained miRNomes revealed colonic adenomas as being an intermediate condition between healthy colon mucosa and adenocarcinoma, which reflected the sequential transition of polyp to cancer in the adenoma-carcinoma transition and confirmed miRNA impairment at early stages of the disease that have already been shown in previous studies [23]. Based on the statistical estimates of differential expression analysis, numerous miRNAs were found to be deregulated along the adenoma-carcinoma sequence. Most of them, including hsa-miR-1246, hsa-miR-215-5p, hsa-miR-31, hsa-miR-135b-5p, and hsa-miR-375, have been previously shown to be deregulated in CRC [16,24,25,26,27], underlining their possible involvement in carcinogenesis. Moreover, in our dataset, hsa-miR-1246 and hsa-miR-215-5p showed the highest correlation with the adenoma-carcinoma transition—the former displayed a gradual increase, while the latter showed a gradual decrease in expression levels along the heathy-adenoma-carcinoma groups, which is partly in line with a previous study by Nagy et al. [23]. Nagy et al. study used microarray (discovery) and RT-PCR (validation) approaches to identify deregulated miRNAs in adenoma to cancer sequence. The major limitation of this study is that they used the same patient group in the discovery and validation steps and hsa-miR-215-5p failed to be replicated due to technical issues, whereas hsa-miR-1246 showed significant upregulation only through the normal to adenoma. In our study, these findings were validated in the independent group of patients, proving robust and early deregulation of hsa-miR-1246 and hsa-miR-215-5p, thereby suggesting their possible involvement in the early stages of carcinogenesis.

It has already been shown that hsa-miR-215-5p is frequently downregulated in CRC tissues [25,26,27] and exhibits tumor suppressor activity through the regulation of EGFR or WNT signaling pathways [26,28]. In the case of hsa-miR-1246, a persistent increase of its expression levels was previously reported in both tissue and plasma samples of CRC [29,30], which agrees with our findings as well. Although the origin of hsa-miR-1246 remains controversial due to sequence overlap with the central region of the RNU2-1 transcript [31], this molecule has been repeatedly reported to function as an oncogene and to promote tumor angiogenesis, growth, migration, invasion, metastasis, and stemness in various cancer types, including CRC [29,31,32,33,34]. Our results are in compliance with the aforementioned reports and showed that inhibition of hsa-miR-1246 reduced viability and migration rates of CRC cells, confirming the oncogenic function of hsa-miR-1246.

Selection of in silico-predicted hsa-miR-1246 target genes was based on the facts acquired from transcriptional and functional parts of this study and involved tumor suppressors participating in a signaling pathway that regulates cell fate determination or cell migration, such as the WNT signaling pathway [19]. The genes *AXIN2* and *CFTR* met these criteria and were selected for further investigation. The *AXIN2* gene is known to play an important role in WNT/β-catenin signaling pathway through phosphorylation and degradation of β-catenin [35]. The *CFTR* gene encodes a chloride channel that controls ion and water secretion as well as absorption in epithelial tissues. Although the exact mechanism is unclear, intestinal tumor tissue analysis of *CFTR* knockout mice showed increased β-catenin levels and revealed a functional implication of *CFTR* in WNT pathway regulation [21]. It was already known that CRC tumors frequently contain mutations in WNT pathway components such as *APC*, *CTNNB1*, and/or *AXIN2* genes, which cause aberrant WNT activation [36]. Recent studies have shown the linkage between the hsa-miR-1246 and the WNT signaling pathway and its function in lung and oral carcinogenesis [37,38]. Our data revealed that inhibition of hsa-miR-1246 enhanced levels of AXIN2 and CFTR tumor suppressor proteins in CRC cells. The luciferase assay verified that this effect is caused by direct 3’UTR-specific interaction between their mRNAs and the seed site of hsa-miR-1246, confirming that both *AXIN2* and *CFTR* genes are targets of hsa-miR-1246. This is partly in line with the results by Chai et al., who reported *AXIN2* as a direct target of hsa-miR-1246 in liver cancer cells [32]. The regulation of these WNT-associated targets by hsa-miR-1246 could also explain the different effect on cell proliferation rate (number colonies formed) in Caco-2 and SW620 cells. This may occur due to uneven deregulation of pathways responsible for cell line proliferation, such as the WNT signaling pathway [19], since it has been previously reported that the SW620 cell line shows stronger WNT signaling than Caco-2 cells [39]. Our results are also supported by the previous observations that AXIN2 and CFTR are frequently downregulated in CRC and are both linked with poor prognosis of the patients [21,40,41,42]. Moreover, it has been shown that inactivating mutations in *CFTR* gene increased the early incidence and progression of colon adenomas [43]. While we do not claim to fully understand the function of hsa-miR-1246, altogether, our data, as well as other studies, provide sufficient evidence of hsa-miR-1246 involvement in early CRC carcinogenesis, possibly, via *AXIN2* and *CFTR* gene expression regulation. The identification of early regulatory changes of hsa-miR-1246 expression has the potential to be further investigated in bigger cohorts for disease monitoring and prognosis application of adenoma progression to carcinoma. Moreover, the impact of hsa-miR-1246 inhibition on cell viability, growth, and migration should be pursued in vivo in future studies to assess its possible therapeutic modality, at least in CRC.

This study has certain limitations that need to be acknowledged. The enrolled CRC patients were not tested for *KRAS* and *BRAF* mutations and other types of molecular phenotyping were not performed. Furthermore, due to the sample size, we were unable to evaluate the association of miRNAs with survival and treatment response; however, we did not have an aim to address these issues in our study design and rather focused on general molecular aspects of the adenoma-carcinoma transition. Additionally, due to sampling time discrepancies, we were not able to perform correlation analysis between tissue and plasma; therefore, future studies are needed. In addition, miR-1246 up-regulation in AP tissue samples did not reflect in AP plasma samples. Polyps tend to have lower ability of exosome production and thus of miR-1246 release into circulation, considering the adenomatous polyps as a precancerous condition [44,45]. Since our results were obtained from transcriptome and functional analyses in cancer tissues and cells lines, the oncogenic potential of miRNA-1246 should be further evaluated in a human organoid model derived from normal colon and adenomatous polyp tissues [46,47]. Nevertheless, we believe that our present study has significance in adding additional knowledge on the role of miRNAs in the adenoma-carcinoma transition.

In conclusion, we showed gradual deregulation of hsa-miR-1246 and hsa-miR-215-5p along healthy control, colon adenoma, and adenocarcinoma groups. We also found that hsa-miR-1246 exhibits an oncogenic effect in vitro with an effect on cell viability, colony formation, and migration in CRC cell lines. Finally, we provided evidence of hsa-miR-1246 being involved in *AXIN2* and *CFTR* gene expression regulation, which might affect the WNT signaling pathway and would be the scope of our future explorations.

## 4. Materials and Methods

### 4.1. Study Population

Tissue and plasma samples from patients with CRC (colorectal cancer) or AP (adenomatous polyps) and HC (healthy control) subjects were prospectively collected at the Department of Gastroenterology and Department of Surgery, Lithuanian University of Health Sciences (Kaunas, Lithuania) during 2011–2014. The specimens of all investigated groups were biopsies taken from affected or healthy colorectal tissues during routine colonoscopy or surgical tumor removal. Biopsies of the CRC group were histologically verified as being colorectal adenocarcinomas. The preneoplastic lesion group was comprised of advanced adenoma patients (conventional adenomas with high-grade dysplasia, adenomas of >1 cm diameter or villous histology). The HC group consisted of healthy subjects who underwent colonoscopy due to positive fecal occult blood test (FOBT), but had no history of previous malignancy and were otherwise healthy. Plasma samples were collected within the three-day period from patient admission to hospital; however, some of the samples were taken the next day after surgery. All patients included in the study were of European descent. Clinical and phenotypic characteristics of subjects investigated in profiling and validation cohorts are presented in Table 1.

### 4.2. RNA Isolation

Total RNA (including small RNA fraction) was extracted from snap-frozen tissue and blood plasma samples using miRNeasy Mini Kit (Qiagen, Hilden, Germany) and miRNeasy Serum and Plasma Kit (Qiagen, Hilden, Germany), respectively, according to the recommendations of the manufacturer. The quality and quantity of the isolated RNA were assessed by Nanodrop 2000 spectrophotometer (Thermo Fisher Scientific, Waltham, MA, USA) or by Agilent 2100 Bioanalyzer (Agilent Technologies, Santa Klara, CA, USA).

### 4.3. Small RNA Library Preparation and NGS

Small RNA libraries were generated with 1 µg RNA input per sample applying a standard TruSeq Small RNA Sample Preparation Kit (Illumina, San Diego, CA, USA) protocol. Agilent 2100 Bioanalyzer (Agilent Technologies, Santa Klara, CA, USA) was used for quality and yield assessment of sequencing libraries. Small RNA libraries were randomly selected, pooled into approximately 24 samples/lane, and sequenced (1 × 50 bp single-end reads) on HiSeq2500 (Illumina, San Diego, CA, USA) NGS platform.

### 4.4. Small RNA-Seq Data Analysis

The generated raw sequencing reads (fastq) were processed by cutadapt v1.9 [48], which was used to trim adapter sequences and low-quality bases (<Q20) as well as discard sequences shorter than 18 nt in length. The processed small RNA-seq reads were assigned to miRBase v21, using quantifier.pl [49] and miraligner.jar [50] for reference miRNAs and isomiRs, respectively. In order to reduce false-positives in isomiR sequence substitutions, a mismatch was considered as a real substitution when the minimum fraction rate of reads having that substitution was equal to 0.25. Additionally, only the isomiRs with uniquely mapped substitutions were kept for downstream analyses. Generated miRNA/isomiR counts were normalized using size factor normalization and variance stabilizing transformation (VST) employing the DESeq2 package [51]. The abundance differences of isomiR variations among the CRC, AP, and HC groups were examined by the Kruskal-Wallis test. Differential expression analyses of miRNAs were performed by employing negative binomial generalized linear models and Wald statistics implemented in the DESeq2 R package. The models were fitted using technical batch and age (centered) as covariates, since the average age was significantly different (*p* < 0.05) between CRC and HC groups. Benjamini and Hochberg correction of false discovery rate (FDR) was used for *p*-value adjustment. The miRNAs with P_FDR_ < 0.01 and absolute value of log2 fold change (log_2_FC) > 0.5 were considered as significantly differentially expressed. Such a log_2_FC and P_FDR_ value threshold was chosen to detect mild changes in expression (0.5 < log_2_FC < 1), which are considerably consistent (P_FDR_ < 0.01) among patients within the group. The results of three-way differential expression analysis were visualized using the volcano3D R package [52]. Spearman’s rank correlation coefficient was used for correlation analysis. The raw sequencing data, as well as miRNA counts, were deposited at the Gene Expression Omnibus (GEO) under the accession number of GSE160432.

### 4.5. RT-qPCR Validation Analysis

Quantification of hsa-miR-1246 and hsa-miR-215-5p expression in tissue samples of CRC, AP, and healthy individuals were analyzed using TaqMan^®^ MicroRNA Assays (Applied Biosystems, Foster City, CA, USA) according to the producer’s recommendations in technical duplicates. MiRNA expression in plasma samples was quantified in two batches—hsa-miR-1246 and hsa-miR-215-5p expression in CRC, and healthy controls plasma samples was quantified by using TaqMan^®^ MicroRNA Assays (Applied Biosystems, Foster City, CA, USA), while expression in AP and the same healthy controls’ plasma samples were analyzed by using TaqMan^®^ Advanced MicroRNA Assays (Applied Biosystems, Foster City, CA, USA). Accordingly, the results of miRNA expression in plasma of CRC and AP groups were analyzed separately. Endogenous hsa-miR-16-5p (tissue and plasma) and hsa-miR-191-5p (plasma) were used as internal normalizers in both tissue and plasma samples [53,54]. The expression levels of hsa-miR-1246 and hsa-miR-215-5p were normalized using ΔCT method [55]. The mean value of duplicate ΔCT values of each sample was submitted to the non-parametric Mann-Whitney U test. The miRNAs with a false discovery rate (FDR) corrected *p* < 0.05 and an absolute value of log2 fold change >1 was considered as significantly differentially expressed.

### 4.6. Target Gene Set Enrichment Analysis

Target gene set enrichment analysis for hsa-miR-1246 and hsa-miR-215 was accomplished using the miTALOS v2 [21] tool. The tool was used to perform overrepresentation analysis of biological pathways with default settings using TargetScan miRNA-target collection and employing Fisher’s exact test as well as Benjamini-Hochberg multiple test correction procedure. Pathways with P_FDR_ < 0.05 and enrichment score (E) > 1 were considered as significantly overrepresented among hsa-miR-1246 and/or hsa-miR-215 target genes.

### 4.7. Cell Culture

Human colorectal adenocarcinoma SW620 (CCL-227™, ATCC^®^, Manassas, VA, USA), Caco-2 (HTB-37™, ATCC^®^, Manassas, VA, USA), HCT116 (CCL-247™, ATCC^®^, Manassas, VA, USA), and gastric adenocarcinoma AGS (CRL-1739™, ATCC^®^, Manassas, VA, USA) cell lines were cultured in Ham's F-12K (Kaighn's) Medium (Gibco, Waltham, MA, USA) supplemented with 10% fetal bovine serum (Gibco, Waltham, MA, USA) and 1% penicillin/streptomycin (100 U/mL penicillin and 100 mg/mL streptomycin, Corning, New York, NY, USA), in a humidified incubator at 37 °C with 5% CO_2_.

### 4.8. Cell Transfection

For colony formation, MTT, wound healing, and luciferase and protein expression assays, reverse transfection with hsa-miR-1246 inhibitor (Assay ID: MH13182, mirVana™, Carlsbad, CA, USA) or mimic (Assay ID: MC13182, mirVana™, Carlsbad, CA, USA) and miR-Control (mirVana™, Carlsbad, CA, USA) (final concentration 100 pmol/mL) was performed using lipofectamine 3000 reagent (Invitrogen, Carlsbad, CA, USA).

### 4.9. Wound Healing Assay

Reverse transfected HCT116 and Caco-2 cells were seeded into a 2-well silicone insert with a defined cell-free gap (Ibidi, Gräfelfing, Germany) for the wound healing assay (4 × 10^4^ cells per well). Before the insert removal, the cells were cultured until reaching 90% confluency. The formed gap was captured at four different time points every 24 h with an inverted light microscope (Olympus IX71, Tokyo, Japan). The ratio between the remaining and the initial size of the wound area was evaluated using Image-J software (Bethesda, MD, USA). At least three independent experiments were performed.

### 4.10. MTT Assay

MTT assay was used for the assessment of the metabolic activity of the cells, which reflects the quantity of viable cells. Reverse transfected SW620 and Caco-2 cells were seeded into a 96-well plate (5000 cells per well). 3-(4,5-dimethylthiazol-2-yl)-2,5-diphenyltetrazolium bromide (MTT; Sigma-Aldrich, Burlington, MA, USA) (final concentration 0.5 mg/mL) was added into each well 72 h after the seeding. Cells were incubated with MTT reagent for another 2 h and, after that, the medium with reagent was removed. Formed formazan crystals were dissolved using dimethyl sulfoxide (DMSO; Carl Roth GmbH + Co., Karlsruhe, Germany) (200 Ul). Light absorbance was detected with Sunrise plate reader (Tecan, Männedorf, Switzerland) at 570 nm wavelength and a reference wavelength at 620 nm. At least three independent experiments were performed.

### 4.11. Colony Formation Assay

Both reverse transfected Caco-2 and SW620 cell lines were seeded into a 6-well plate (250 cells per well). After 2 weeks of incubation with complete medium, the cells were washed with PBS, fixed with 10% formaldehyde (Sigma Aldrich, Burlington, MA, USA) for 20 min, and stained with 1% crystal violet (Alfa Aesar by Thermo Fisher Scientific, Kandel, Germany) for 15 min at room temperature. The number of colony-forming units was calculated using ImageJ software (Bethesda, MD, USA) in three independent experiments.

### 4.12. Target Prediction

Putative target genes, having 8-mer binding sites for hsa-miR-1246, were retrieved from the TargetScan v7.2 [56] database. Since the hsa-miR-1246 showed increased expression levels (oncogenic) in CRC, the targets were selected based on their function in carcinogenesis, i.e., targets having tumor-suppressive function were chosen for further investigation.

### 4.13. Protein Extraction and Western Blot

Reverse transfected SW620 and Caco-2 cells were seeded in 6-well plates (1 × 10^6^ cells per well) for protein expression experiments; 72 h after transfection, cells were lysed using 1× radioimmunoprecipitation assay (RIPA) buffer (Abcam, Cambridge, UK) containing phosphatase and protease inhibitor cocktail (Sigma Aldrich, Burlington, MA, USA). Total protein concentration was evaluated using Pierce BCA Protein Assay Kit (Thermo Fisher Scientific, Waltham, MA, USA). The extracted protein was fractionated by SDS-PAGE using 4–12% Bis-Tris Plus Mini Gels and transferred to 0.45 µm PVDF membrane. PVDF membranes were blocked using WesternBreeze^TM^ Blocker/Diluent (Part A and Part B) (Thermo Fisher Scientific, Waltham, MA, USA) at room temperature for 1 h. Antibodies directed against AXIN2 (1:1000 dilution; Cat. No. ab109307; Abcam, UK), CFTR (1:500 dilution; Cat. No. ab2784, Abcam, UK), and GAPDH (0.4 µg/mL concentration; Cat. No. AM4300; Ambion by Thermo Fisher Scientific, Waltham, MA, USA) were used. Protein signals were detected and visualized using ChemiDocTM XRS+ System (Bio-Rad, Hercules, CA, USA) and ImageLab Software v5.2.1 (Bio-Rad, Hercules, CA, USA). GAPDH protein was used as an endogenous control. At least three independent experiments were performed.

### 4.14. Dual Light Luciferase Assay

Wild-type (WT) and mutant (MT) seed region sites of hsa-miR-1246 containing 3’UTR sequence of target genes were constructed and cloned into pMIR-REPORT-Luciferase vector (Invitrogen, Carlsbad, CA, USA) (Figure 3B). Constructed insert sequences were verified by Sanger sequencing using BigDye Terminator v3.1 kit (Applied Biosystems, Foster City, CA, USA) and 3500 Series Genetic Analyzer (Applied Biosystems, Foster City, CA, USA). A previously well-established workflow for dual luciferase assay using AGS cells was applied for this experiment [57,58]. Cells were co-transfected with hsa-miR-1246 mimic and miR-Control, pMIR-REPORT-Luciferase-WT vector, pMIR-REPORT-Luciferase-MT vector, and pMIR-REPORT-ß-galactosidase control vector (Invitrogen, Carlsbad, CA, USA). Luciferase activity was detected after 48 h of incubation by the Dual-Light™ Luciferase & β-Galactosidase Reporter Gene Assay System (Invitrogen, Carlsbad, CA, USA) and normalized with ß-galactosidase activity using Tecan Genios Pro (Tecan, Männedorf, Switzerland). At least three independent experiments were performed.

### 4.15. Statistical Analysis

All data from the functional experiments in CRC cell lines are given as means ± standard deviation (SD) of at least three independent experiments. Data between groups were compared using a two-tailed Student’s *t*-test for the normally distributed data or the Mann-Whitney U test for the not normally (shown by Shapiro-Wilk test) distributed data. All statistical calculations were performed with R Studio software version v3.6.0 (Boston, MA, USA). False discovery rate (FDR) corrected *p* < 0.05 was considered statistically significant.

## Figures and Tables

**Table 1:** Clinical and phenotypic information of profiling and validation cohorts. Categorical variables are summarized as absolute numbers and percentages (in parentheses), while age (quantitative variable) is summarized as mean and standard deviation. To test for differences among groups, Chi squared test was used for categorical variables, while Kruskal-Wallis test was used for quantitative variables.

		Profiling Cohort (*n* = 72)				Validation Cohort (*n* = 120)			
		AP (*n* = 20)	CRC (*n* = 20)	HC (*n* = 32)	*p* Value	AP (*n* = 40)	CRC (*n* = 40)	HC (*n* = 40)	*p* Value
Sex	Male	8 (40%)	10 (50%)	12 (37.5%)		20 (50%)	23 (57.5%)	20 (50%)	
	Female	12 (60%)	10 (50%)	20 (62.5%)	0.662	20 (50%)	17 (42.5%)	20 (50%)	0.74
Age (mean, sd)		63 ± 8	68 ± 8	58 ± 12	0.012	63 ± 10	63 ± 10	57 ± 15	0.18
Smoking	Yes	8 (40%)	2 (10%)	4 (12.5%)		1 (2.5%)	3 (7.5%)	5 (12.5%)	
	No	10 (50%)	11 (55%)	23 (71.9%)		27 (67.5%)	12 (30%)	26 (65%)	
	Unknown	2 (10%)	7 (35%)	5 (15.6%)	0.028	12 (30%)	25 (62.5%)	9 (22.5%)	0.00001
Type	Tubular adenoma	13 (65%)	-	-		11 (27.5%)	-	-	
	Adenoma	5 (25%)	-	-		28 (70%)	-	-	
	Tubulovillous adenoma	2 (10%)	-	-		-	-	-	
	Papiloadenoma	-	-	-		1 (2.5%)	-	-	
	Adenocarcinoma	-	20 (100%)	-		-	40 (100%)	-	0.004
Localization	Caecum	-	2 (10%)	-		2 (5%)	1 (2.5%)	-	
	Ascending colon	1 (5%)	5 (25%)	-		3 (7.5%)	4 (10%)	-	
	Transverse colon	3 (15%)	2 (10%)	-		-	-	-	
	Descending colon	6 (30%)	1 (5%)	-		5 (12.5%)	6 (15%)	-	
	Sigmoid colon	5 (25%)	5 (25%)	-		20 (50%)	2 (5%)	-	
	Rectum	5 (25%)	5 (25%)	-		7 (17.5%)	27 (67.5%)	-	
	#N/A	-	-	-		3 (7.5%)	-	-	
Stage of CRC	0	-	8 (40%)	-		-	18 (45%)	-	
	I	-	1 (5%)	-		-	2 (5%)	-	
	II	-	4 (20%)	-		-	4 (10%)	-	
	III	-	4 (20%)	-		-	14 (35%)	-	
	IV	-	3 (15%)	-		-	2 (5%)	-	0.451

## Data Availability

The raw sequencing data as well as miRNA counts have been deposited at the Gene Expression Omnibus (GEO) under the accession number of GSE160432. All other data presented in this study are included within the paper and its Supplementary Information or are available upon request from the corresponding author.

## Supplementary Materials

The following are available online at https://www.mdpi.com/article/10.3390/ijms23042107/s1.
