# Supplementary material for: miRNome Profiling and Functional Analysis Reveal Involvement of hsa-miR-1246 in Colon Adenoma-Carcinoma Transition by Targeting AXIN2 and CFTR
Source: Int J Mol Sci. 2022 Feb 14;23(4):2107. doi: 10.3390/ijms23042107 (PMC8876010; doi:10.3390/ijms23042107)
Supplement: Supplementary file 1 [file ijms-23-02107-s001.zip › Supplementary Figures (1).pdf]

SUPPLEMENTARY INFORMATION

Human miRNome profiling and functional analysis reveal hsa-miR-1246 involvement in adenoma-carcinoma sequence via WNT signaling pathway

Juzenas et al.

SUPPLEMENTARY FIGURES

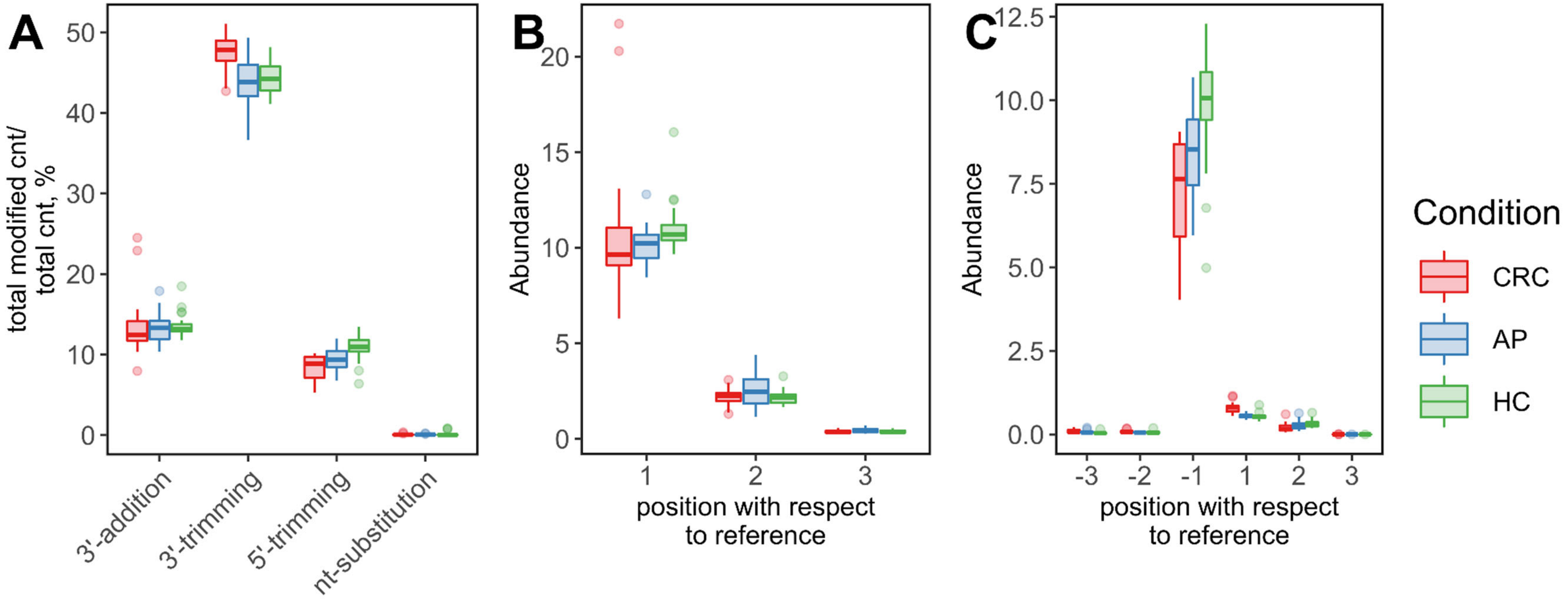

Supplementary Figure S1. Distributions of isomiR modification types across colorectal cancer (CRC), adenomatous polyps (AP) patients and healthy controls (HC). Boxplots showing: **A)** abundance of modification types; **B)** abundance of 3’ addition modification; **C)** abundance of 5’ trimming modification.

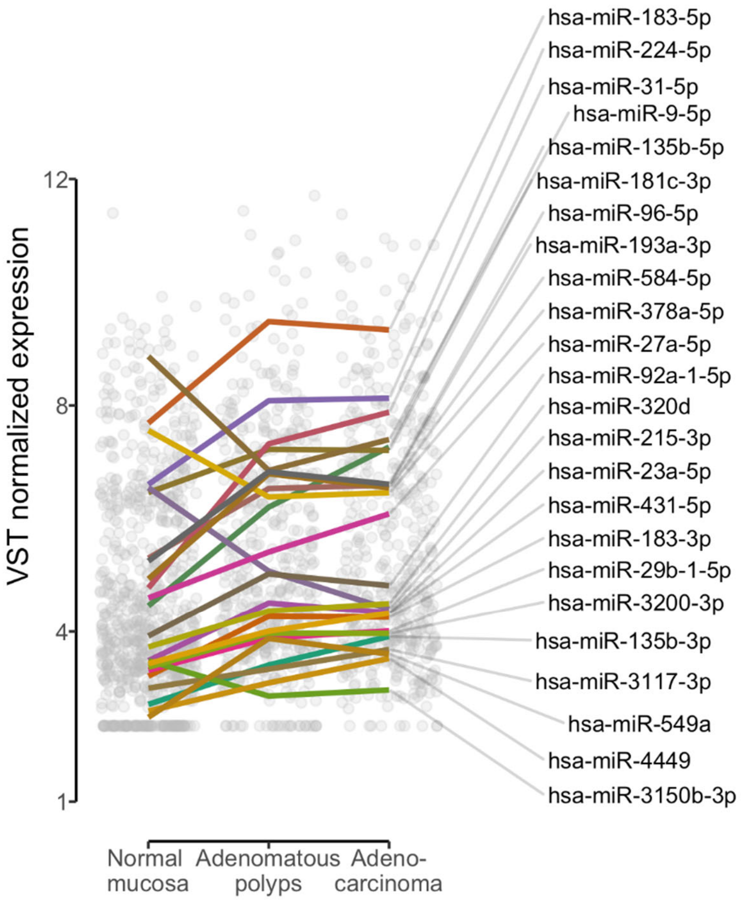

Supplementary Figure S2. Gradual expression of miRNAs in polyp-to-cancer sequence. Moderately correlating (Sprearman's 0.5 < r < 0.7) miRNAs with the stages of healthy-to-polyp-to-cancer sequence. Spearman correlation analysis was performed on variance stabilization transformed (VST) counts.

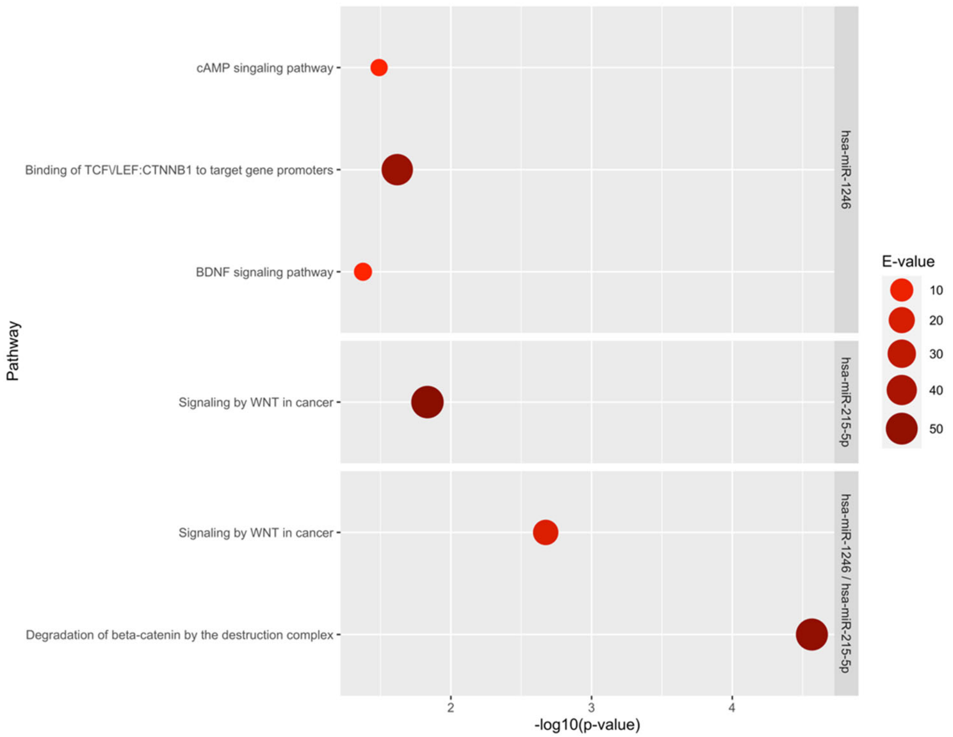

Supplementary Figure S3. Target gene set enrichment analysis (GSEA) results. Analysis was performed using miTALOS v2 using default settings. Pathways with $P_{FDR} < 0.05$ and enrichment score (E-value) > 1 were considered as significantly overrepresented among hsa-miR-1246 and/or hsa-miR-215 target genes.
